# Novel Approach to Cluster Patient-Generated Data Into Actionable Topics: Case Study of a Web-Based Breast Cancer Forum

**DOI:** 10.2196/medinform.9162

**Published:** 2018-11-29

**Authors:** Josette Jones, Meeta Pradhan, Masoud Hosseini, Anand Kulanthaivel, Mahmood Hosseini

**Affiliations:** 1 Health Informatics BioHealth Informatics Department Indiana University, Indianapolis Indianapolis, IN United States; 2 Indiana Biosciences Research Institute Indianapolis, IN United States; 3 Purdue School of Science and Technology Purdue University, Indianapolis Indianapolis, IN United States

**Keywords:** data interpretation, natural language processing, patient-generated information, social media, statistical analysis, infodemiology

## Abstract

**Background:**

The increasing use of social media and mHealth apps has generated new opportunities for health care consumers to share information about their health and well-being. Information shared through social media contains not only medical information but also valuable information about how the survivors manage disease and recovery in the context of daily life.

**Objective:**

The objective of this study was to determine the feasibility of acquiring and modeling the topics of a major online breast cancer support forum. Breast cancer patient support forums were selected to discover the hidden, less obvious aspects of disease management and recovery.

**Methods:**

First, manual topic categorization was performed using qualitative content analysis (QCA) of each individual forum board. Second, we requested permission from the Breastcancer.org Community for a more in-depth analysis of the postings. Topic modeling was then performed using open source software Machine Learning Language Toolkit, followed by multiple linear regression (MLR) analysis to detect highly correlated topics among the different website forums.

**Results:**

QCA of the forums resulted in 20 categories of user discussion. The final topic model organized >4 million postings into 30 manageable topics. Using qualitative analysis of the topic models and statistical analysis, we grouped these 30 topics into 4 distinct clusters with similarity scores of ≥0.80; these clusters were labeled Symptoms & Diagnosis, Treatment, Financial, and Family & Friends. A clinician review confirmed the clinical significance of the topic clusters, allowing for future detection of actionable items within social media postings. To identify the most significant topics across individual forums, MLR demonstrated that 6 topics—based on the Akaike information criterion values ranging from −642.75 to −412.32—were statistically significant.

**Conclusions:**

The developed method provides an insight into the areas of interest and concern, including those not ascertainable in the clinic. Such topics included support from lay and professional caregivers and late side effects of therapy that consumers discuss in social media and may be of interest to clinicians. The developed methods and results indicate the potential of social media to inform the clinical workflow with regards to the impact of recovery on daily life.

## Introduction

Health care is currently undergoing transformation by capitalizing on information technology and patient-consumer engagement and activation through health information technology such as patient portals and mHealth apps. Consumer engagement is assumed to strengthen providers’ abilities to tailor their care to the consumers’ needs, preferences, and abilities. The increasing use of smartphones, mobile apps, and remote monitoring devices, coupled with providers’ deployments of electronic health records, patient portals, and secure messaging, offers innovative ways to connect patients and providers and to strengthen consumers’ engagement in their health and well-being [1]. In addition, health consumers have embraced social media, enabling them to share and discuss how they manage their health and well-being with others with similar health issues. These social media and mHealth apps generate important data outside the health care settings and, when shared with providers, expand the depth, breadth, and continuity of information available to optimize health care and outcomes.

Despite the proliferation of social media use, such as blogs and forums, little is known about the scope and quality of information shared, or the purposes that social media sites serve for consumer decisional and support needs [2]. Social media retains large amounts of valuable information about consumers’ contextual and environmental (day-to-day) factors while managing their health and well-being; such issues form a major foundation of human health. However, analyzing those free-text data to discover these *hidden* aspects of health consumers’ lives and understand their health information needs beyond those routinely addressed by health care providers is challenging [3].

This study explores approaches for analyzing the social media data and extract potential valuable information on managing health and well-being beyond the context of health care. As it is known that breast cancer patients and survivors often join social media to fulfill their information needs and discuss their daily challenges and concerns, all or not related to health [4], using those venues is apparent. Some concerns might not be shared with health care providers for many reasons. For example, patients might think it is not necessary to discuss the topic, may feel embarrassed about the issue, or they do not even know there is a problem [5,6]. Hence, we explored ways to discover issues that are not commonly shared but are important for the overall health and well-being.

## Methods

### Overview

**Figure 1:**
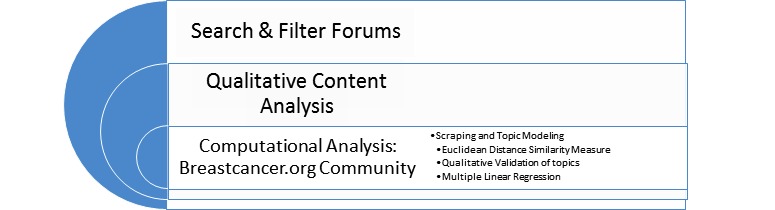
Overview of the methods used to analyze the study content.

First, manual topic categorization was performed using qualitative content analysis (QCA) of each individual forum board. Second, we requested permission from the Breastcancer.org Community for a more in-depth analysis of the postings. In addition, natural language processing (NLP) and statistical modeling approach were used to cluster >4 million postings into manageable topics. Finally, topic modeling was performed using open source software, followed by multiple linear regression (MLR) analysis to detect highly correlated topics among the different website forums. The methodology is outlined in Figure 1.

### Manual Categorization of Posts

A Google search was performed for breast cancer forum websites. Selection criteria were active websites (having posts in the week of search) and in the English language. Each website must have at least 5000 members or have a minimum of 50,000 posts in total, and the posts on the site must be organized into categories. Among the resulting 20 websites, 5 were included on the basis of the selection criteria. These 5 remaining websites contained 4,901,516 posts organized in 211 forums (Table 1). The forum posts were further manually analyzed for consensus among the team members.

Team members were assigned to review the titles of 211 forums across the 5 forum websites and organize them into 4 main top-level categories and 16 subcategories correlating to several domains from the report on the social determinants of health published by the Institute of Medicine [12]. The quantities of posts belonging to each category and subcategory were calculated by the forum that the posts belonged to. The 3 most dominant subcategories across all websites were as follows: Treatments (1.49/4.90 million, 30.5%, posts), Diagnosed–Psychosocial Support–Similar Patients (1.34/4.90 million, 27.3%, posts), and Diagnosed–Psychosocial Support–Life (0.83/4.90 million, 16.9%, posts). After the posts were categorized, the research team iteratively validated (with a random sample of 20 posts from each forum) and consolidated the initial categorization, assuring the quality and correctness of the method.

**Table 1:** Breast cancer websites explored.

Website name	Site URL	Country	Forums	Threads	Posts	Members
Breastcancer.org Community [7]	community.breastcancer.org	US^a^	80	121,688	3,608,324	153,620
Breast Cancer Care [8]	forum.breastcancercare.co.uk	UK^b^	54	36,949	782,486	N/A^c^
Susan G Komen Foundation: Message Board [9]	apps.komen.org/Forums	US	24	44,175	354,592	26,883
Triple Negative Breast Cancer: Forums [10]	forum.tnbcfoundation.org	US	17	9641	100,706	123,427
No Surrender Breast Cancer Foundation [11]	nosurrenderbreastcancersupportforum.com	US	36	2443	55,498	5549

^a^US: United States.

^b^UK: United Kingdom.

^c^N/A: not applicable.

### Data Extraction, Natural Language Processing, and Statistical Modeling

Data from a public breast cancer internet discussion forum were extracted, cleaned, and processed; multiple approaches merging NLP with statistical modeling were implemented for knowledge discovery. In addition, off-the-shelf products were used to develop and streamline the analytical approach to cluster most-occurring topics of discussions. The methodology developed revealed several topics that may be of importance for care planning and, thus, need to be incorporated in the electronic health record. In addition, advanced text mining will be a foundation for predictive modeling of consumers’ health information needs and provide interactive solutions.

### Extracting (Scraping) Forum Data

Postings from the Breastcancer.org Community website were selected for further analyses, as this website contained the highest number of total posts (3.61/4.90 million 73.6%, posts across all 5 websites selected in this study). Permission was obtained from the Web administrators to download and analyze all the data logged in the site.

The Breastcancer.org Community site includes 80 main forums organized by the site administrators into 9 sections. Users self-select in which forum, and thus in which section, a post that they make will go. To capture information within the forum posts, an in-house scraping tool in the *PHP Hypertext Preprocessor* language was developed by a team member. Forum metadata along with the actual posts were extracted; the text within the different posts was aggregated into 80 text files each corresponding to a forum. The files were named based on the forum ID number. The data were saved in the JavaScript Object Notation format. Multimedia Appendix 1 shows the forum names along with the number of threads and posts in each forum.

### Applying Topic Modeling

Topic models provide a simple way to analyze large volumes of unlabeled text. A “topic” consists of a cluster of words that frequently occur together. Using contextual clues, topic models can connect words with similar meanings and distinguish between uses of words with multiple meanings. One of the leading approaches used for topic modeling is Latent Dirichlet allocation (LDA), which is one of the most popular methods in NLP [13]. LDA represents a document as a distribution of “topics,” where a topic is itself a distribution over words (and may or may not be similar to a forum topic). Looping through each word in every document, the LDA algorithm assigns every word to a temporary topic in a semirandom manner and iteratively updates topic assignments. For each word, its topic assignment is subsequently updated based on 2 criteria as follows: (1) the prevalence of the word across all topics and (2) the prevalence of the topics within the documents.

The Machine Learning Language Toolkit (MALLET) open source tool (University of Massachusetts, Amherst, MA, USA) [14] was used to execute the LDA algorithm on the data to extract the main topics. MALLET is a Java-based tool developed at the University of Massachusetts Amherst, which is used for the analysis of data in a textual format such as document classification, clustering, topic modeling, information extraction, and other machine learning apps. After scraping the forum data and saving into 80 files representing each forum, the files were imported into the MALLET tool. MALLET generates two tab-delimited text files as a result of algorithm execution. One file contains the topic ID, and the words related to that topic (aka the topic keywords; Table 2).

MALLET was run iteratively, customized to generate 15 topics, 20 topics, and 30 topics, respectively. Topic labels were added by consensus of the research team based on the semantics of the word cluster. Some topic labels in different sets of topics were identical based on the semantic similarity, but the topic words and strength are different for each of the 3 sets generated. No new topics were generated at the third iteration; the MALLET categorization of 30 word baskets was used for further analysis.

For each iteration and each file, the topic composition and corresponding LDA strength were computed, providing us a way to infer the latent structure of the text file. The resulting output is a topic ID-by-text file matrix known as a file-feature set (Table 3). The first column shows the name of the file; the rest of the columns are best considered as (topic-ID, topic-strength) pairs. For example, it is noted that file F100 has a Topic 12 strength of 0.275 (27.5%). For each document, there are as many of these pairs as there are topics, although only the top 5 topics for each file and the first 4 files are shown for brevity.

**Table 2:** The partial table of topics generated by Machine Learning Language Toolkit in the 30-topic model, with interpretations (the list goes on up to the 30th topic; only 3 are shown for brevity).

Machine Learning Language Toolkit topic identifier	Topic label	Topic keywords
1	Diagnostic testing and waiting for results	breast biopsy cancer lump results ultrasound benign surgeon mammogram doctor mri waiting back mammo good radiologist feel pain left i'm
2	Side effects of inflammation and its treatment	breast ibc skin symptoms pain rash red cancer nipple biopsy infection diagnosed antibiotics swollen treatment left specialist redness swelling lymph
3	Positive results after recurrence	chemo stage years cancer treatment nodes onc tumor triple negative taxol positive rads year diagnosed node recurrence congratulations lymph radiation

**Table 3:** A portion of the file-feature set generated by Machine Learning Language Toolkit software (the list goes on up to the 80th file and 30th topic; values were truncated for brevity of display).

File identifier^a^	Topic ID^b^	Strength^c^	Topic ID	Strength	Topic ID	Strength	Topic ID	Strength	Topic ID	Strength
F100	12	0.275	18	0.269	2	0.251	5	0.06	7	0.053
F102	2	0.542	18	0.136	7	0.087	12	0.056	1	0.04
F104	2	0.315	14	0.118	1	0.104	7	0.09	20	0.043
F105	2	0.295	11	0.25	6	0.213	7	0.067	14	0.042

^a^Scraped forum file.

^b^Topic identifier: Machine Learning Language Toolkit-generated topics.

^c^Weight of topic in the file.

### Statistical Analyses

The output from MALLET assigned weight scores (ie, topic-strength) to each topic-ID within each file. Statistical analysis was carried out (1) to understand the similarity across the feature sets and files and (2) to identify the topics that are most relevant to patients with breast cancer. Euclidean Distance Similarity Measures (EDSM) were computed to evaluate the similarity across the files based on their weight scores for each topic. Equation (1) is an example of how each file and its feature vectors were assigned, using file F100 as an example.

F100 = [Topic6(.33), Topic1(.28), Topic9(.20), Topic2(.08), Topic0(.05), Topic3(.04), Topic12(.01), Topic4(.01), Topic n(weight m)…]

1

The EDSM between all potential file pairs were computed on the basis of Equation (2):

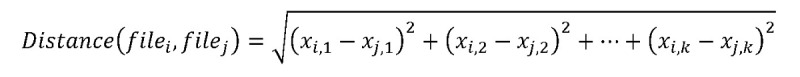



Where *i* and *j* are identifiers for each file pair, *k* is the total number of topics in the dataset (ie, 15, 20, or 30 topics), and *x* is the topic weight score in each file.

To identify the most relevant topics in the dataset, an MLR analysis was performed on all files. The MLR analysis was computed using the R Statistical Package [15]. The MLR analysis identified the topics that seem to be most relevant within and across the forums in the study. The equation model (Equation 3) for the MLR used is:

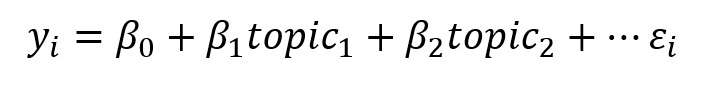



Where *topic_1_*, *topic_2_* are the weight scores of the topics in the files; *ε*_i_ is the error in the model; β_0_ is the intercept; *β*_1_, *β*_2_ are the coefficients for the *topic_1_*, *topic_2_*, respectively, computed by the model; and y_i_ is the outcome (dependent variable) for each file *i*.

## Results

### Manual Categorization of Posts

Among the 5 sites studied, Breastcancer.org Community presented the majority of the total post volume. From all websites analyzed for post counts, 73.6% (3.61/4.90 million) of posts were from Breastcancer.org Community; hence, we selected this site for further exploration.

### Manual Categorization

**Figure 2:**
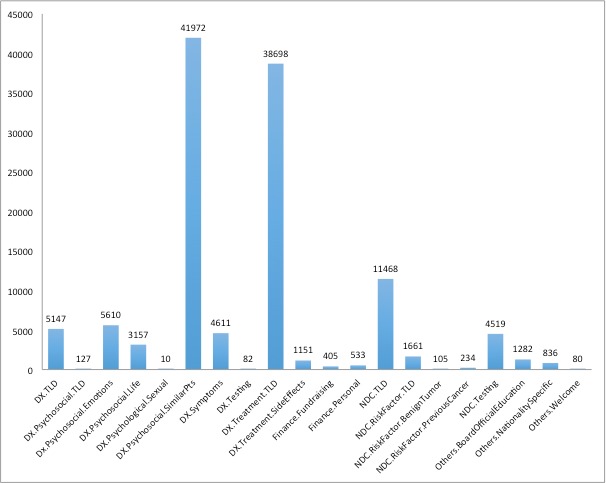
Distribution of qualitative content analysis-generated categories according to the number of forum threads that each manual category possesses. DX: Diagnosed; TLD: Top-Level Domain; NDC: Not Diagnosed but Concerned.

Initially, the research team performed via QCA a manual categorization of topics discussed in the 5 selected public websites. The popularity distribution of the manually generated categories, as discussed in the Methods section, was assessed by the number of posts made in the forums. For example, the qualitatively generated categories *Diagnosed—Psychosocial— Similar Patients* have an overall popularity of 41,972 posts. The forum categorization was either general or granular depending on the forum structure. The distribution of qualitative categories across the threads on the Breastcancer.org Community website is visualized in the figures below by frequencies of category popularity (Figure 2); 20 of our QCA-generated categories mapped to forums on breastcancer.org. The *x*-axis shows the QCA-assigned category names.

### Data Extraction, Natural Language Processing, and Statistical Modeling

#### Data Extraction

The data for all 80 forums on the Breastcancer.org Community website were successfully extracted into 80 files, each containing all communications posted over its respective forum.

#### Natural Language Processing: Topic Modeling

As mentioned earlier, exhaustion was reached at baskets of 30 cooccurring words. The remainder of the analyses will be only for these topics. All machine-generated topics were assigned topic labels based on the semantics of the word cluster and validated by a domain expert (clinical); the topic ID was equated with the term key. MALLET assigned an LDA strength to each topic indicating its overall dominance across all forum files that were analyzed. Two example topics, IDs #8 and #29, are listed in Table 4 (below), along with the authors’ labels for these topics. Multimedia Appendix 2 provides a full list of generated topics from this model as well as the authors’ semantic interpretations. Each file represents the text of one forum, and topic-strength pairs for the strongest five topics per MALLET LDA analysis of that file are found to the right of the file’s ID. For any file, the strength across all 30 topics will always add up to 1.00.

MALLET also correlated the topic relationship strengths between all files based on their topics. Strengths assigned to document-topic pairs by MALLET ranged from almost zero (<0.000001) up to 0.796. The maximum theoretical possible strength for a single file-topic pair would be 1.00. Multimedia Appendix 3 provides a list of the top 5 correlated topics of each file.

#### Statistical Analysis: Euclidean Distance Similarity Measures

**Figure 3:**
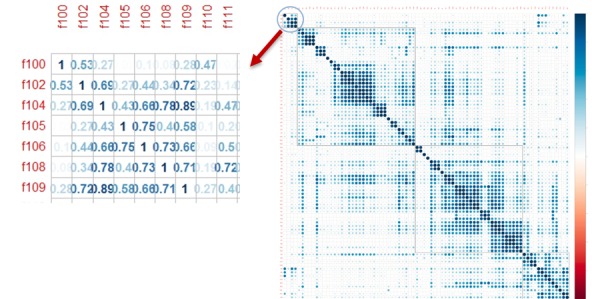
File-file similarity matrix.

EDSM were calculated to find the similarity between files. Figure 3 shows the file-file similarity matrix and a subset of the similarity matrix. The files are mirrored across both axes and ordered by their alphabetical file name (with F100 being first and F99 being last). Darker cells indicate that the files were more similar. File 109 has a similarity measure of 0.89 with file 104; similarly, file 108 has a similarity measure of 0.78 with file 104.

Table 5 illustrates the similarity measures among the file pairs with a similarity score ≥0.8.

**Table 4:** Topics #8 and #29 with Latent Dirichlet allocation strengths author topic label interpretations.

Topic identifier	Latent Dirichlet allocation strength	Topic words	Authors’ topic label
8	1.38724	cancer chemo years feel life family mom time support things breast people treatment don’t husband care friends diagnosed talk mother	Hope, love, family, and friends
29	0.19954	hair book pink survivor happy deb health country president shirley obama congratulations cats article eye mammo fumi beth beautiful vote	Daily living and breast cancer

**Table 5:** Top scored file-file similarity measures.

File identifier	Associated files (similarity score)
F102	F133 (0.85), F144 (0.91), F152 (0.97), F116 (0.95)
F104	F109 (0.89), F142 (0.81), F150 (0.82), F27 (0.89)
F108	F132 (0.94), F137 (0.86), F145 (0.97), F5 (0.90), F71 (0.93), F88 (0.86), F96 (0.97)
F109	F104 (0.89), F142 (0.89), F127 (0.85)
F110	F26 (0.80)
F111	F132 (0.8), F68 (0.87)
F112	F47 (0.92), F93 (0.94)
F113	F139 (0.89), F55 (0.87)
F133	F102 (0.85), F135 (0.86), F145 (0.90), F5 (0.87), F71 (0.94), F96 (0.94), F88 (0.87)

A cursory review of the files with high similarity also revealed clinical relevance and connection. For example, files F108, 132, and 145, while in different discussion categories on the website, all discuss the diagnosis, treatment, and potential side effects from the treatments and also discuss living with different stages and types of breast cancer. In addition, F96 has a high similarity (97%) with F108, which is devoted to the breast cancer type known as invasive ductal carcinoma. F112 discusses more specific genetic risks of breast cancer (BRCA1 or BRCA2 positive), while F47 (similarity 92%) chats about more general risks. F93 at first glance seems not related (Comments, Suggestions, Feature Requests), but reading the postings revealed the need for more information and social support for users who find out that they are *at risk* for breast cancer.

**Figure 4:**
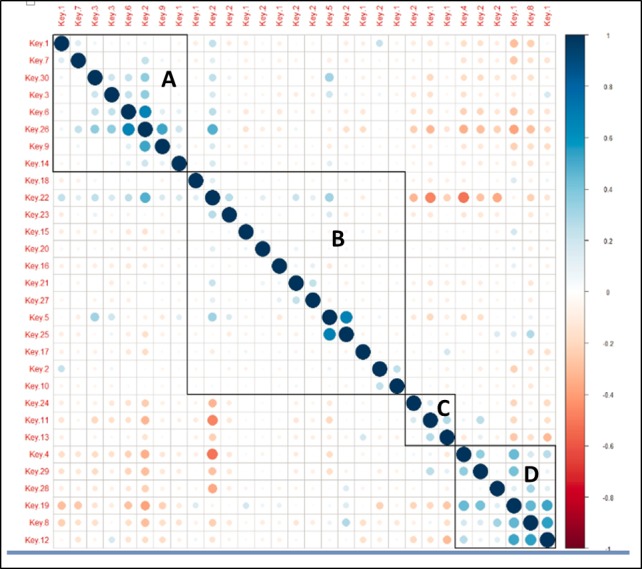
Topic-topic similarity matrix.

Figure 4 shows 4 clusters of highly correlated computationally modeled topics. Each cluster is labeled with a letter, and topics are labeled as *keys*. The topics with their consensus labels based on the semantic meaning of the word baskets are as follows:

Cluster A: Symptoms & Diagnosis

- *Topic1*: Diagnostic testing and waiting for result
- *Topic 7*: Genetic risk and testing
- *Topic 30*: Symptoms and diagnosis of recurrence
- *Topic 3*: Positive results after recurrence
- *Topic 26*: Positive results after treatment for recurrence
- *Topic 9*: Diagnostic and treatment observation for recurrence
- *Topic 14*: Medical drug treatment and long-term effects

Cluster B: Treatment

- *Topic18*: Chemotherapy side effects and change of treatment
- *Topic 22*: General feeling over time
- *Topic 23*: Medical or drug treatment and side effects
- *Topic15*: Physical activities during and after chemo
- *Topic 20*: Side effects of breast cancer treatment
- *Topic16*: Surgical treatments while in remission
- *Topic 21*: Lingering side effects while in remission
- *Topic27*: Surgical reconstruction during remission
- *Topic 5*: Prognosis about relapse or recurrence
- *Topic 25*: Support from caregivers and medical team for recovery long term
- *Topic 17*: Nutrition and supplements
- *Topic 2*: Side effect of inflammation and its treatment
- *Topic 10*: Radiation side effects and duration of the effects.

Cluster C: Financial

- *Topic 24*: Financial issues over time
- *Topic 11*: Forum-related discussion for support from people in similar circumstances
- *Topic 13:* Looking for clinical research and trials

Cluster D: Friends & Family

- *Topic 4*: Friends and fun
- *Topic 29*: Everyday life and breast cancer
- *Topic 28*: Spirituality and religion
- *Topic 19*: feeling back to normal
- *Topic 8*: Hope, love, family, and friends
- *Topic 12*: Feeling positive and support

**Table 6:** Most significant topics identified via multiple linear regression analysis.

Topic identifier	Topic label	Akaike information criterion values
21	Lingering side effects while in remission	−642.75
18	Chemotherapy side effects and change of treatment	−641.98
10	Radiation and side effects	−633.17
7	Genetic risk and testing	−620.41
25	Support from caregiver and medical team for recovery long term	−571.78
11	Looking for support from people in similar circumstances	−412.32

As can be deduced from the semantic labeling, each cluster describes a theme: cluster A is related to risk factors, diagnosis, and potential risk of recurrence, whereas cluster B describes different treatments and their side effects in the short and long term. Cluster C and D are less clinically and more oriented to patient contextual factors (ie, those that are typically ascertainable only outside of the clinic encounters).

#### Statistical Analyses: Multiple Linear Regression Analysis

Finally, MLR analysis was performed to identify the most significant topics (keys) across the 4 abovementioned clusters. The topics were arranged in a descending order based on the Akaike information criterion value, the most appropriate measure for the methodology. The most significant topics identified by the model were: Topic21> Topic18> Topic10> Topic7> Topic25> Topic11. Table 6 reports the most significant topics discussed among the forum participants, along with the respective topic labels assigned by the authors.

## Discussion

### Principal Findings

It is well known that many users share information online daily. Forum posts, blogs, or other social media activity reveal a rich diary of everyday life. Health information is revealed explicitly when an individual communicates about their well-being or when they ask for guidance, information on a very specific health issue, treatment, and other related topics. Our goal was to explore a method to enhance our ability to collect and interpret information from those social media sources. Our methodology allowed us to organize 4 million plus postings into 30 topics, consequently clustered into 4 groups.

The popularity of QCA-generated categories (as measured by the number of posts in their associated forums) showed a logarithmic-linear (*log-lin)* distribution, strongly suggesting that a few QCA-generated categories are disproportionately gravitated toward user self-selection, while most topics receive comparatively little attention. Moreover, it is of great interest that topic modeling analysis via MALLET showed that the overall LDA strengths of each topic among the forum documents (as seen in Figure 3) also followed a log-*lin* type of distribution, allowing for the same type of conclusion in objectively quantifying content with regards to the MALLET-generated topics.

In addition, a modest level of correlation was observed between the strongest (via MLR analysis) MALLET topics and the strength (by user posting) of manual QCA-generated categories. Topic 11, *looking for support from people in similar circumstances*, is almost semantically identical to the QCA-generated category of *Diagnosed—Psychosocial—Similar Patients.* The latter category encompassed 34.4% (41,972/121,688 of all threads on the Breastcancer.org Community website. Topics 10 and 18 (*Radiation and side effects* and *Chemotherapy side effects and change of treatments*, respectively), meanwhile, correlate in general to the manually generated category of *Diagnosed—Treatment* (top-level domain), which covered 38,698 threads on the site. Topic 7 (*genetic risk and testing*) is semantically similar to the QCA-generated category of *Not Diagnosed But Concerned—Testing*, noted in 4519 site user threads.

The computationally assigned importance of Topic 11 when combined with its equally significant manually generated category correlate demonstrates the need for health personnel to take into account the contextual (nonclinical) factors unlikely to be captured in conventional medical documentation and not supported by conventional clinic-based information technology resources. In particular, a greater emphasis on information-mediated psychosocial interventions is supported by the results of this research.

The computational topic modeling analysis via MALLET also demonstrated topics that did not arise via manual category generation. These topics, in particular Topic 21 (*lingering side effects while in remission)* and Topic 25 (*support from caregiver and medical team for recovery long term*), mirror breast cancer survivorship instead of the disorder itself. Therefore, it is suggested that computational topic modeling software such as MALLET is useful in future research on large bodies of patient-generated text and can generate topics similar in quality to those generated by expert QCA; furthermore, this type of software can detect significant but *hidden* topics (such as social and daily living issues dealt with during survivorship) that are not otherwise detectable when only the forum labels given by a site are analyzed qualitatively.

Visual analysis of the file-file (ie, forum-forum) similarity matrix (see Figure 4) shows a particular concentration of similar files across the diagonal axis, indicating that files numbered with close numbers tended to be more similar in content. This observation actually does strengthen the case for using computational topic modeling software such as MALLET because closely numbered files in the study at hand tended to originate from forums that resided in identical categories on the Breastcancer.org Community website. Similarly, topics that closely correlated with each other were noted to have clinically significant correlates. It is important to note that many of these correlates may not have been intuitive at face value but were more explainable with the clinical expertise.

Overall, the research team was able to gain significant insight into the daily lives, clinical and otherwise, of patients affected by breast cancer; the onus to support survivors of breast cancer was also revealed. Furthermore, the research performed generated significant support for the use of computational topic modeling software such as MALLET to analyze patient-generated information for nonclinical issues revealed by patients with breast cancer over relevant disease-specific online forums.

### Limitations and Future Considerations

The data could be better annotated in metadata-facilitated context as opposed to being in a purely free-text format; the granularity of the ontology in which the data are stored can be improved in future research. In particular, having the posts traceable to unique anonymized users would be of assistance. To achieve this granularity, forums in the future can be scraped in a manner that preserves the HTML source code of their content; structured information could be then extracted from the HTML. The granularity of time, if extractable from the HTML, could potentially facilitate the generation of individual patient records and potentially even allow for the capability for analyzing patient narratives in a longitudinal (time-wise) fashion.

Detailed analysis of similarity measures between files and clustering methods is an important part of potential future research and will require thorough analysis by clinical and patient health experts. This process is very time consuming and is out of the scope of this study as our goal in this work is to present a method for modeling a data in a meaningful format. Future work will focus more on further analysis of data to identify hidden relationships between files and topics that might reveal hidden aspects of breast cancer patients’ challenges in their real life.

### Conclusions

The importance of patient-generated data (including patient-generated information via online communications) is growing among scholars because of their value in identifying hidden aspects of patients’ challenges and concerns. This study provides a reasonable amount of insight into the areas of interest or concern that patients with breast cancer discuss in social media and may need to be addressed to optimize patient disease and health management.

## Appendix

Multimedia Appendix 1 The 80 forums and their groupings on the Breastcancer.org Community website.

Multimedia Appendix 2 File-feature set.

Multimedia Appendix 3 List of topics generated by MALLET in the 30 topic model.

## Abbreviations

EDSM: Euclidean Distance Similarity Measures
LDA: Latent Dirichlet allocation
MALLET: Machine Learning Language Toolkit
MLR: multiple linear regression
NLP: natural language processing
QCA: qualitative content analysis
