# Supplementary material for: Novel Approach to Cluster Patient-Generated Data Into Actionable Topics: Case Study of a Web-Based Breast Cancer Forum
Source: JMIR Med Inform. 2018 Nov 29;6(4):e45. doi: 10.2196/medinform.9162 (PMC6293240; doi:10.2196/medinform.9162)
Supplement: Multimedia Appendix 1 [file medinform_v6i4e45_app1.pdf]

This is a Multimedia Appendix to a full manuscript published in the J Med Internet Res. For full copyright and citation information see http://dx.doi.org/10.2196/jmir.9162-150423-1

MULTIMEDIA APPENDIX I.

The 80 forums and their groupings on the Breastcancer.org Community website

Section	File number	Forum	Threads
**Welcome to Breastcancer.org**	131	Information and resources for new patients, and new members	15
	135	Acknowledging and honoring our Community	82
**Not Diagnosed but Concerned**	83	Not Diagnosed but Worried	12183
	62	Waiting for Test Results	4844
	148	Benign Breast Conditions	207
	47	High Risk for Breast Cancer	1745
**Tests, Treatments, Side Effects**	5	Just Diagnosed	5487
	147	Diagnosed and Waiting for Test Results	161
	6	Help Me Get Through Treatment	2855
	91	Surgery - Before, During, and After	5998
	44	Breast Reconstruction	9384
	82	Living Without Reconstruction After a Mastectomy	1052
	64	Lymphedema	4510
	136	Pain	266
	69	Chemotherapy - Before, During, and After	6026
	70	Radiation Therapy - Before, During and After	2787
	78	Hormonal Therapy - Before, During and After	5692
	79	Complementary and Holistic Medicine and Treatment	2412
	121	Alternative Medicine	438
**Connecting With Others Who Have a Similar Diagnosis**	68	DCIS (Ductal Carcinoma In Situ)	3197
	111	Micro-invasive DCIS that is HER2 positive	51
	95	LCIS (Lobular Carcinoma In Situ)	478

	96	IDC (Invasive Ductal Carcinoma)	2387
	81	IBC (Inflammatory Breast Cancer)	1268
	71	ILC (Invasive Lobular Carcinoma)	1013
	137	Less Common Types of Breast Cancer	84
	132	Mixed Type Breast Cancer	47
	142	Breast Cancer with Another Diagnosis or Comorbidity	78
	108	Stage I Breast Cancer	1016
	145	Stage II Breast Cancer	133
	67	Stage III Breast Cancer	3375
	8	Stage IV and Metastatic Breast Cancer ONLY	19880
	105	Not Diagnosed With a Recurrence or Metastases but Concerned	1820
	106	Just Diagnosed With a Recurrence or Metastasis	684
	88	Second or Third Breast Cancer	361
	80	HER2+ (Positive) Breast Cancer	1600
	72	Triple-Negative Breast Cancer	1866
	112	BRCA1 or BRCA2 Positive	328
	51	Male Breast Cancer	171
	77	Palliative Therapy/Hospice Care	177
**Day-to-Day Matters**	150	Sex Matters	33
	152	Share Your Experiences Of Life After A Breast Cancer Diagnosis	32
	156	Your Furry Friends	3
	120	Bone Health and Bone Loss	148
	133	Coping with Holidays, Special Days and Anniversaries	62
	113	Employment, Insurance, and Other Financial Issues	569
	102	Relationships, Emotional Crises, Anxiety, and Depression	1207
	58	Fitness and Getting Back in Shape	675
	86	Healthy Recipes for Everyday Living	651
	31	Humor and Games	1799
	26	Recommend Your Resources	563
	149	BCO Free-Cycle: Give or trade items related to breast cancer	22

	73	Clinical Trials, Research Studies, News, and Study Results	4829
**Recovery, Renewal, Hope**	7	Moving Beyond Cancer	2972
	84	Growing our Friendships After Treatment	474
	23	Biographies and Inspiring Stories	294
	38	Prayers and Spiritual Inspiration	784
**Support Community Connections**	144	For Family and Caregivers of Members with STAGE IV Diagnosis	211
	153	Member Matchup	6
	16	For Caregivers, Family, Friends and Supporters	1340
	138	Australian and New Zealand Breast Cancer Survivors	30
	55	Canadian Breast Cancer Survivors	641
	139	International, Non-English Speakers with Breast Cancer	11
	98	African Americans with Breast Cancer	166
	141	Latinas/Hispanics with Breast Cancer	6
	99	Singles with Breast Cancer	167
	76	LGBT with Breast Cancer	179
	27	Young With Breast Cancer	726
	109	Middle Age (40-60(ish) Years with Breast Cancer	231
	104	Older than 60 Years with Breast Cancer	206
	85	High Risk of Recurrence or Second Breast Cancer	237
	56	Commemorating Loved Ones	130
	61	BC.org Chatters	309
	34	Get Togethers	368
	63	Walks, Runs and Fundraising Events for Breastcancer.org	311
**Site News and Announcements**	93	Comments, Suggestions, Feature Requests	496
	90	Mod Announcements, Breastcancer.org News, Blog Entries, Podcasts	40
**Advocacy and Fund-Raising**	114	Fundraising Opportunities for Breastcancer.org	16
	110	Advocacy	244
	100	Donated to Breastcancer.org in honor of...	64
