# Supplementary material for: Novel Approach to Cluster Patient-Generated Data Into Actionable Topics: Case Study of a Web-Based Breast Cancer Forum
Source: JMIR Med Inform. 2018 Nov 29;6(4):e45. doi: 10.2196/medinform.9162 (PMC6293240; doi:10.2196/medinform.9162)
Supplement: Multimedia Appendix 2 [file medinform_v6i4e45_app2.pdf]

This is a Multimedia Appendix to a full manuscript published in the J Med Internet Res. For full copyright and citation information see http://dx.doi.org/10.2196/jmir.9162-150423-1

MULTIMEDIA APPENDIX III.

File-feature set. Each file represents the text of one forum, and topic-strength pairs for the strongest five topics per MALLET LDA analysis of that file are found to the right of the file's ID. For any file, the strength across all 30 topics will always add up to 1.00.

File ID	Topic ID	Strength	Topic ID	Strength	Topic ID	Strength	Topic ID	Strength	Topic ID	Strength
F100	12	0.275	18	0.269	2	0.251	5	0.06	7	0.053
F102	2	0.542	18	0.136	7	0.087	12	0.056	1	0.04
F104	2	0.315	14	0.118	1	0.104	7	0.09	20	0.043
F105	2	0.295	11	0.25	6	0.213	7	0.067	14	0.042
F106	2	0.311	1	0.148	11	0.143	19	0.131	3	0.06
F108	2	0.3	1	0.219	4	0.101	3	0.099	14	0.071
F109	2	0.34	14	0.11	1	0.1	7	0.094	5	0.071
F110	13	0.319	12	0.296	2	0.191	6	0.051	15	0.039
F111	4	0.374	2	0.214	3	0.168	1	0.132	7	0.037
F112	15	0.439	2	0.278	1	0.089	7	0.045	12	0.033
F113	12	0.544	2	0.273	7	0.047	1	0.026	10	0.019
F114	12	0.513	2	0.22	13	0.109	5	0.078	7	0.04
F120	14	0.475	2	0.181	7	0.106	13	0.073	9	0.064
F121	9	0.286	2	0.238	13	0.206	4	0.059	7	0.052
F131	12	0.405	13	0.234	2	0.113	15	0.061	1	0.056
F132	1	0.249	2	0.214	4	0.202	3	0.103	15	0.073
F133	2	0.42	5	0.223	18	0.123	7	0.113	12	0.046
F135	2	0.343	18	0.137	7	0.133	12	0.105	1	0.092
F136	2	0.295	17	0.217	14	0.097	7	0.08	6	0.06
F137	1	0.247	2	0.201	6	0.173	3	0.127	4	0.115
F138	2	0.278	5	0.2	7	0.143	1	0.109	12	0.096
F139	5	0.76	2	0.058	12	0.037	15	0.03	1	0.029
F141	5	0.312	20	0.22	12	0.132	15	0.113	2	0.113
F142	2	0.318	14	0.166	1	0.086	7	0.077	11	0.072
F144	2	0.464	18	0.182	19	0.155	1	0.082	7	0.056
F145	2	0.317	1	0.255	3	0.109	11	0.068	14	0.056
F147	1	0.293	2	0.28	7	0.094	6	0.09	16	0.05
F148	6	0.615	2	0.183	15	0.05	4	0.044	7	0.032
F149	17	0.311	12	0.25	2	0.173	7	0.095	16	0.085
F150	2	0.4	14	0.252	7	0.082	10	0.061	5	0.056
F152	2	0.492	7	0.147	18	0.081	12	0.056	1	0.054

**F153**	12	0.377	2	0.175	7	0.085	1	0.083	20	0.058
**F156**	2	0.328	5	0.303	12	0.149	18	0.126	7	0.046
**F16**	2	0.466	18	0.21	1	0.138	7	0.055	12	0.038
**F23**	2	0.327	1	0.138	18	0.119	5	0.085	7	0.075
**F26**	13	0.235	2	0.197	12	0.184	9	0.086	7	0.078
**F27**	2	0.387	1	0.161	14	0.071	15	0.067	7	0.062
**F31**	5	0.493	2	0.231	7	0.154	18	0.043	12	0.037
**F34**	5	0.294	7	0.234	2	0.207	12	0.111	1	0.059
**F38**	18	0.573	2	0.222	7	0.088	5	0.056	12	0.019
**F44**	10	0.542	2	0.231	7	0.092	17	0.056	12	0.02
**F47**	15	0.353	2	0.242	6	0.15	4	0.066	7	0.042
**F5**	1	0.333	2	0.33	4	0.092	6	0.059	7	0.056
**F51**	2	0.29	6	0.2	1	0.102	15	0.062	12	0.061
**F55**	2	0.255	12	0.223	1	0.093	7	0.086	10	0.063
**F56**	18	0.464	2	0.289	7	0.064	5	0.061	19	0.046
**F58**	7	0.424	2	0.223	9	0.093	10	0.056	8	0.053
**F6**	2	0.338	16	0.249	1	0.098	7	0.092	14	0.05
**F61**	2	0.256	18	0.255	12	0.079	1	0.079	7	0.07
**F62**	6	0.467	2	0.266	1	0.1	7	0.061	4	0.027
**F63**	12	0.33	5	0.219	7	0.19	2	0.169	13	0.055
**F64**	8	0.499	2	0.247	7	0.126	12	0.04	17	0.026
**F67**	2	0.396	11	0.17	7	0.1	1	0.078	16	0.038
**F68**	4	0.555	2	0.282	7	0.046	1	0.037	6	0.036
**F69**	16	0.411	2	0.307	7	0.115	1	0.063	3	0.03
**F7**	2	0.364	5	0.194	7	0.109	12	0.091	18	0.046
**F70**	20	0.357	2	0.299	7	0.103	1	0.056	4	0.047
**F71**	2	0.263	1	0.241	15	0.104	6	0.064	4	0.063
**F72**	3	0.3	2	0.296	1	0.12	7	0.065	13	0.037
**F73**	13	0.638	2	0.114	12	0.059	7	0.042	9	0.032
**F76**	2	0.369	5	0.268	12	0.125	7	0.065	18	0.037
**F77**	2	0.424	18	0.324	19	0.105	7	0.07	12	0.044
**F78**	14	0.521	2	0.253	7	0.086	13	0.03	1	0.022
**F79**	9	0.382	2	0.198	13	0.195	7	0.068	14	0.063
**F8**	2	0.365	19	0.341	7	0.092	12	0.045	11	0.024
**F80**	3	0.482	2	0.279	7	0.068	14	0.045	1	0.028
**F81**	2	0.263	6	0.241	20	0.213	1	0.082	19	0.05
**F82**	17	0.452	2	0.289	7	0.1	10	0.08	12	0.04
**F83**	6	0.584	2	0.255	7	0.048	15	0.03	1	0.026
**F84**	5	0.419	2	0.276	7	0.15	18	0.05	12	0.041
**F85**	2	0.252	15	0.169	4	0.08	1	0.08	3	0.08
**F86**	9	0.67	2	0.13	7	0.112	13	0.037	5	0.026

**F88**	2	0.304	1	0.181	4	0.118	3	0.075	11	0.075
**F90**	12	0.324	13	0.224	2	0.175	7	0.065	18	0.06
**F91**	2	0.299	17	0.249	10	0.104	7	0.096	1	0.091
**F93**	12	0.464	2	0.336	7	0.069	13	0.039	4	0.025
**F95**	15	0.347	2	0.244	4	0.174	6	0.089	7	0.042
**F96**	2	0.297	1	0.292	4	0.099	3	0.076	7	0.057
**F98**	2	0.285	16	0.13	5	0.097	18	0.094	7	0.076
**F99**	2	0.461	5	0.205	7	0.108	12	0.064	18	0.05

(…to 30 topics)
