# Supplementary material for: Novel Approach to Cluster Patient-Generated Data Into Actionable Topics: Case Study of a Web-Based Breast Cancer Forum
Source: JMIR Med Inform. 2018 Nov 29;6(4):e45. doi: 10.2196/medinform.9162 (PMC6293240; doi:10.2196/medinform.9162)
Supplement: Multimedia Appendix 3 [file medinform_v6i4e45_app3.pdf]

This is a Multimedia Appendix to a full manuscript published in the J Med Internet Res. For full copyright and citation information see http://dx.doi.org/10.2196/jmir.9162-150423-1

MULTIMEDIA APPENDIX II.

List of topics generated by MALLET in the 30 topic model. The consensus interpretations of the topics are noted as topic labels to the left, and the topic keywords along with overall LDA strength of each topic are shown to the right. The displayed LDA strengths are MALLET-generated for that topic across all forum files and do not always correlate with the strengths found via multiple regression analysis.

Topic Label	Topic ID	LDA Strength	Topic Keywords (Basket)
**Diagnostic testing and waiting for results**	1	0.34502	breast biopsy cancer lump results ultrasound benign surgeon mammogram doctor mri waiting back mammo good radiologist feel pain left i'm
**Side effects of inflamation and its treatment**	2	0.1486	breast ibc skin symptoms pain rash red cancer nipple biopsy infection diagnosed antibiotics swollen treatment left specialist redness swelling lymph
**Positive results after recurrence**	3	0.16302	chemo stage years cancer treatment nodes onc tumor triple negative taxol positive rads year diagnosed node recurrence congratulations lymph radiation
**Friends and fun**	4	0.21757	love year great man live marin i'm show friends meet post men birthday nice favorite fun thread hope watch hair
**Prognosis about relapse/recurrence and potential treatment**	5	0.29097	mets pain bone scan hope liver onc stage treatment good news scans results months i'm pet luck brain hear week
**diagnostic testing and confirmation**	6	0.31034	chemo cancer treatment tumor surgery surgeon lumpectomy biopsy stage nodes grade diagnosed diagnosis node test positive oncotype results information oncologist
**genetic risks and testing**	7	0.25689	cancer risk breast lcis brca family genetic history test mri years testing diagnosed women tested high mutation gene tamoxifen age
**hope, love, family, friends**	8	1.38724	cancer chemo years feel life family mom time support things breast people treatment don't husband care friends diagnosed talk mother
**diagnosis and treatment options for recurrence**	9	0.18974	dcis cancer radiation risk lumpectomy grade invasive treatment biopsy mastectomy margins diagnosis surgeon tamoxifen mri recurrence breast stage pathology rads
**radiation treatment (duration and side effects)**	10	0.25876	radiation rads treatment skin rad treatments chemo breast weeks week area finished onc pain day side start chest started oncologist
**looking for support from people in similar circumstances**	11	1.3873	post people women site cancer thread stage forum breast agree information posts link read don't support page find list add

**feeling positive and support**	12	0.6806	i'm feel great good happy time year back day wonderful feeling hugs days people news love it's post positive hope
**looking for clinical research and trial**	13	0.31412	cancer breast patients study women research cells drug treatment risk drugs clinical tumor article studies years cell cancers disease trial
**medical (drug) treatment and long term effects**	14	0.2025	herceptin chemo treatment heart positive onc year cancer weeks stage tumor taxol tch oncologist treatments years recurrence test trial negative
**physical activities during and after chemo**	15	0.29143	exercise weight walk walking back running chemo run week yoga pounds training body started miles lbs gym lose weeks eat
**surgical treatment while in remission**	16	0.25678	bra wear bras mastectomy reconstruction breast flat forms form chest recon prosthesis size don't comfortable breasts wearing feel side women
**nutrition and suplements**	17	0.20384	cancer eat diet food soy oil vitamin Multimedia Appendixs chemo alternative taking good natural foods organic tea read estrogen water sugar
**Chemotherapy side effects and change of treatment**	18	0.19378	chemo hair treatment taxol weeks port day days taxotere started pain onc treatments week effects side wig luck feel start
**feeling back to normal**	19	1.50473	good day time back don't thought put it's i'm work you're i've made life make didn't today night people long
**side effects of breast cancer treatment**	20	0.10157	arm lymphedema sleeve therapist swelling compression hand lymph wear risk glove nodes garments pain binney treatment fluid massage breast it's
**side effects while in remission**	21	0.16468	surgery pain surgeon mastectomy drains weeks lumpectomy arm drain hospital days home removed bmx nodes side reconstruction node day recovery
**general feelings over time**	22	2.40411	i'm don't it's good time years hope i've find back told lot found long make months doctor didn't ago thing
**medical (drug) treatment and side effects**	23	0.2484	tamoxifen years taking arimidex pain effects side onc tamox estrogen hot femara months flashes started women chemo cancer drug i'm
**Financial issues over time**	24	0.27058	insurance health pay cancer company care work year coverage breast money medicare medical hospital plan treatment state job paid bill
**support from care giver and medical team for recovery long term**	25	0.12625	great cancer mets xeloda love hope wonderful hugs good news happy working tumor onc progression family stable zometa oncologist enjoy
**positive result after treatment for recurrence**	26	1.01657	breast cancer surgery mastectomy radiation feel surgeon time decision luck women lumpectomy nodes good back removed tumor left small weeks
**surgical reconstruction during in remission**	27	0.25671	surgery implants reconstruction breast diep implant side exchange flap skin weeks nipple tissue pain surgeon time fat good feel expanders

**spirituality and religion**	28	0.15557	god prayers love pray family peace lord dear prayer hospice praying bless thoughts strength heart mother comfort day hope jesus
**every day and breast cancer**	29	0.19954	hair book pink survivor happy deb health country president shirley obama congratulations cats article eye mammo fumi beth beautiful vote
**symptoms and diagnosis of recurrence**	30	0.21851	cancer pain scan chemo ilc years onc mri bone recurrence back mets oncologist nodes tumor stage node test scans pet
